# Supplementary material for: The transcription factor OCT6 promotes the dissolution of the naïve pluripotent state by repressing Nanog and activating a formative state gene regulatory network
Source: Sci Rep. 2024 May 7;14:10420. doi: 10.1038/s41598-024-59247-5 (PMC11074312; doi:10.1038/s41598-024-59247-5)
Supplement: Supplementary file 1 — Supplementary Information 1. [file 41598_2024_59247_MOESM1_ESM.docx]

# The transcription factor OCT6 promotes the dissolution of the naïve pluripotent state by repressing *Nanog* and activating a formative state gene regulatory network.

#

# Supplementary Figures

###

### Figure S1


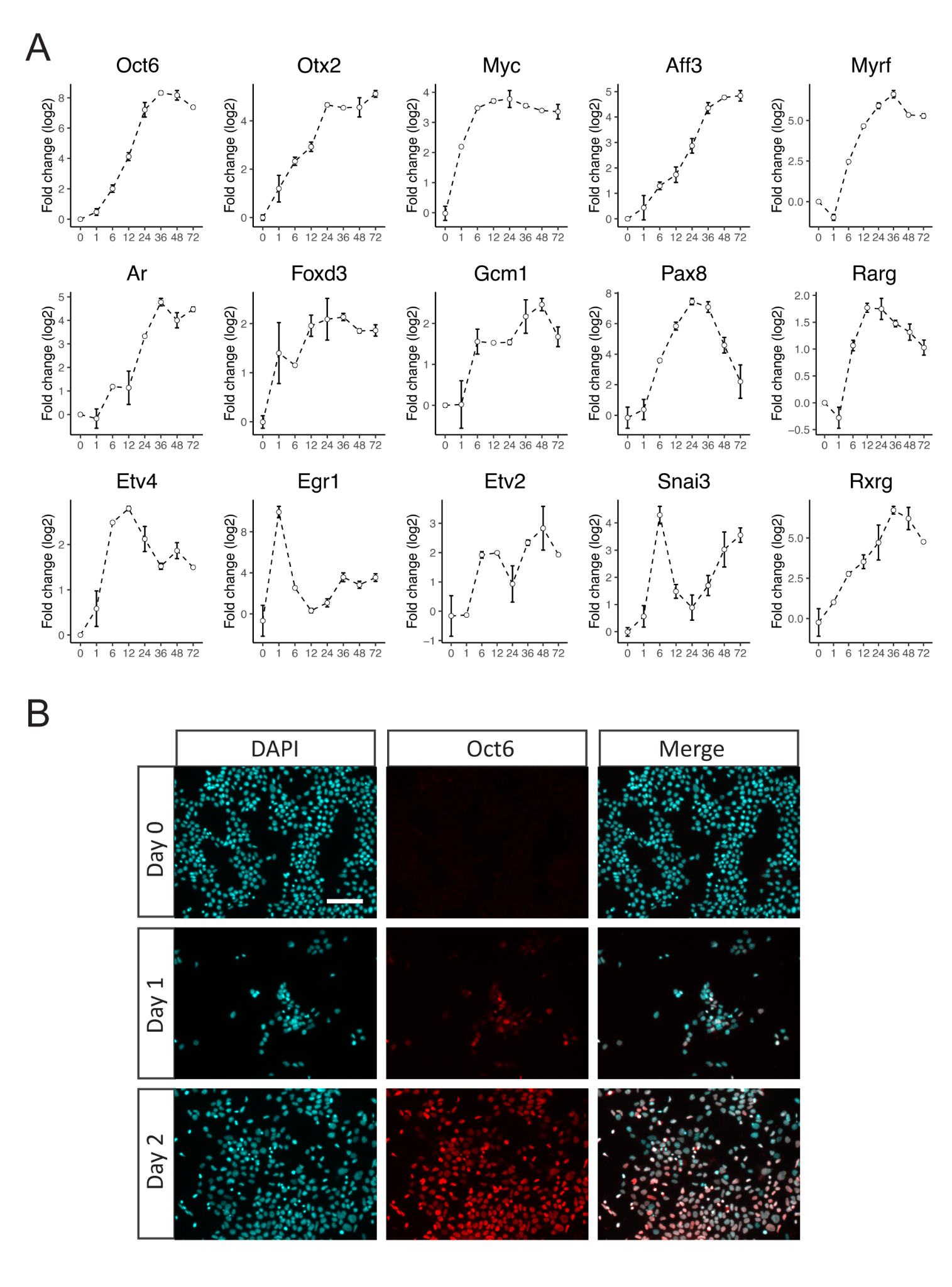


### Figure S2


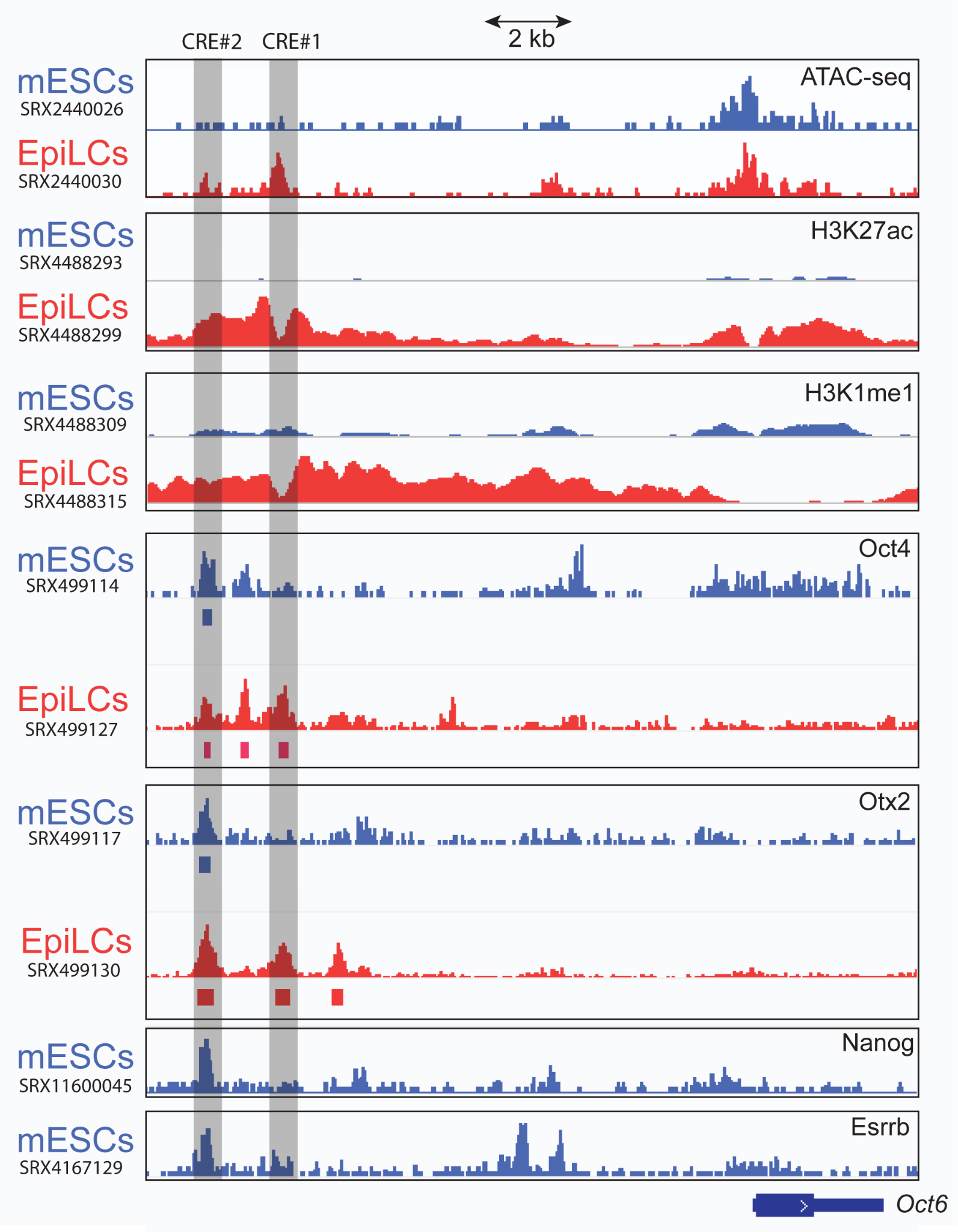


### Figure S3


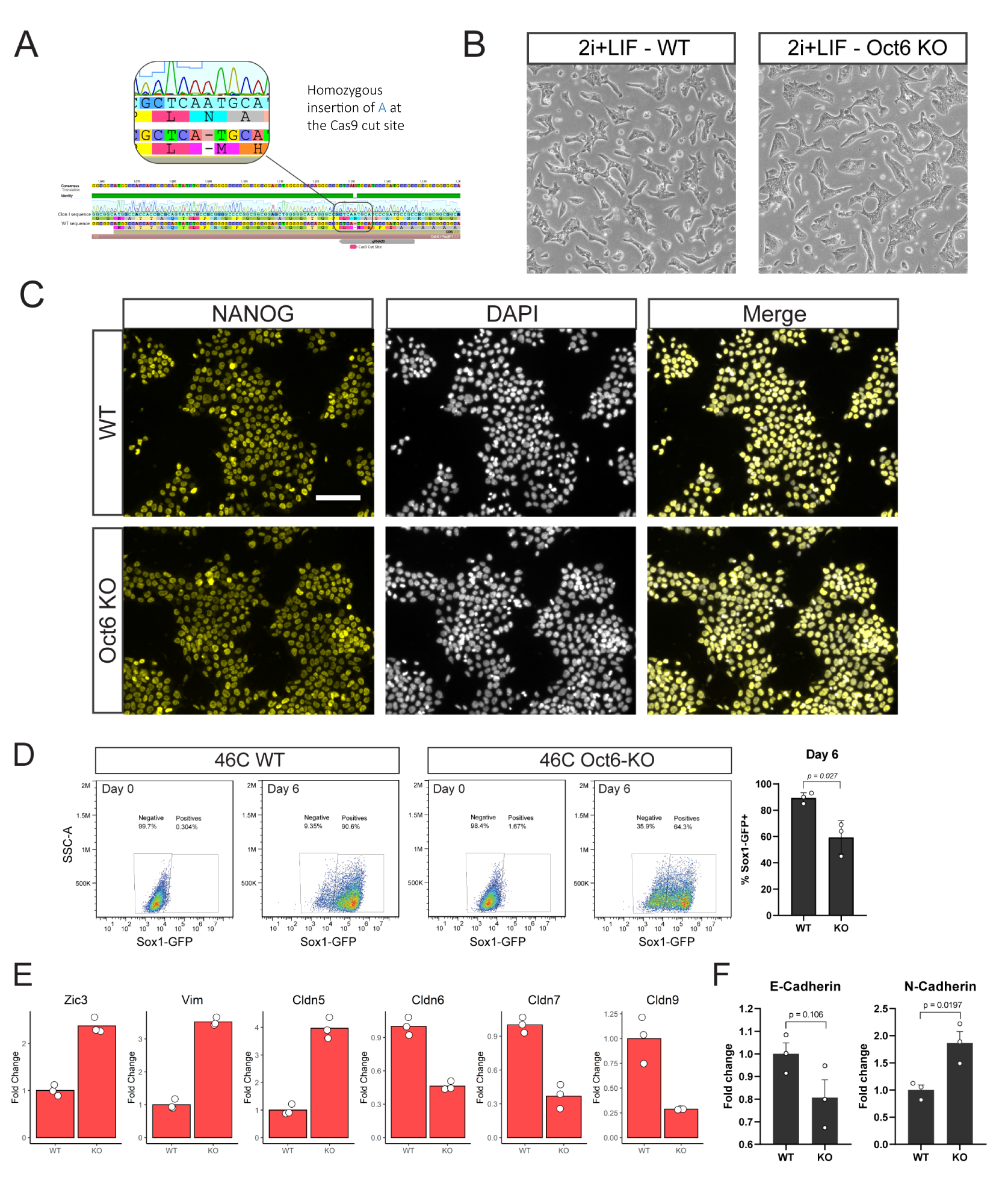


### Figure S4


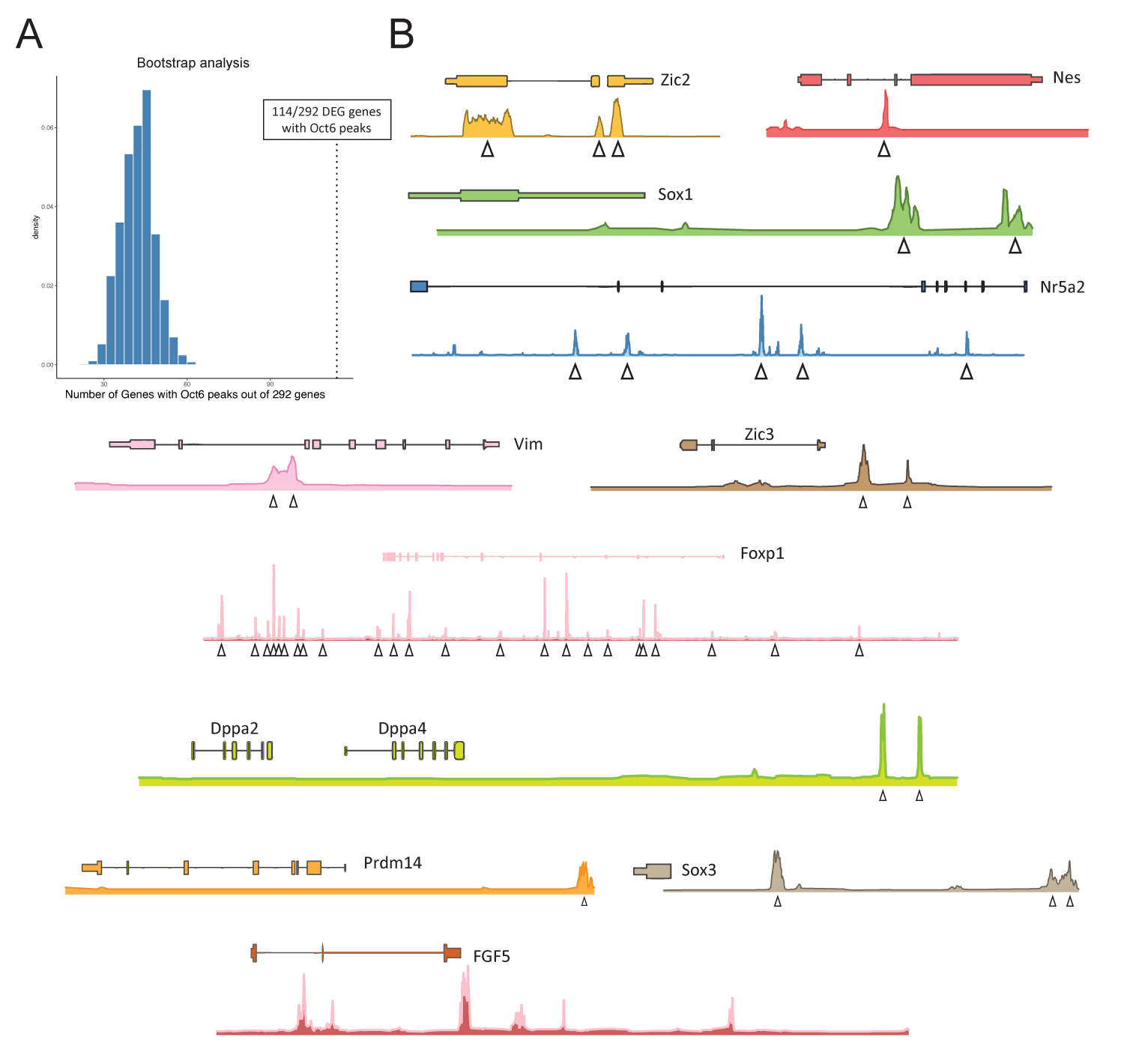


### Figure S5


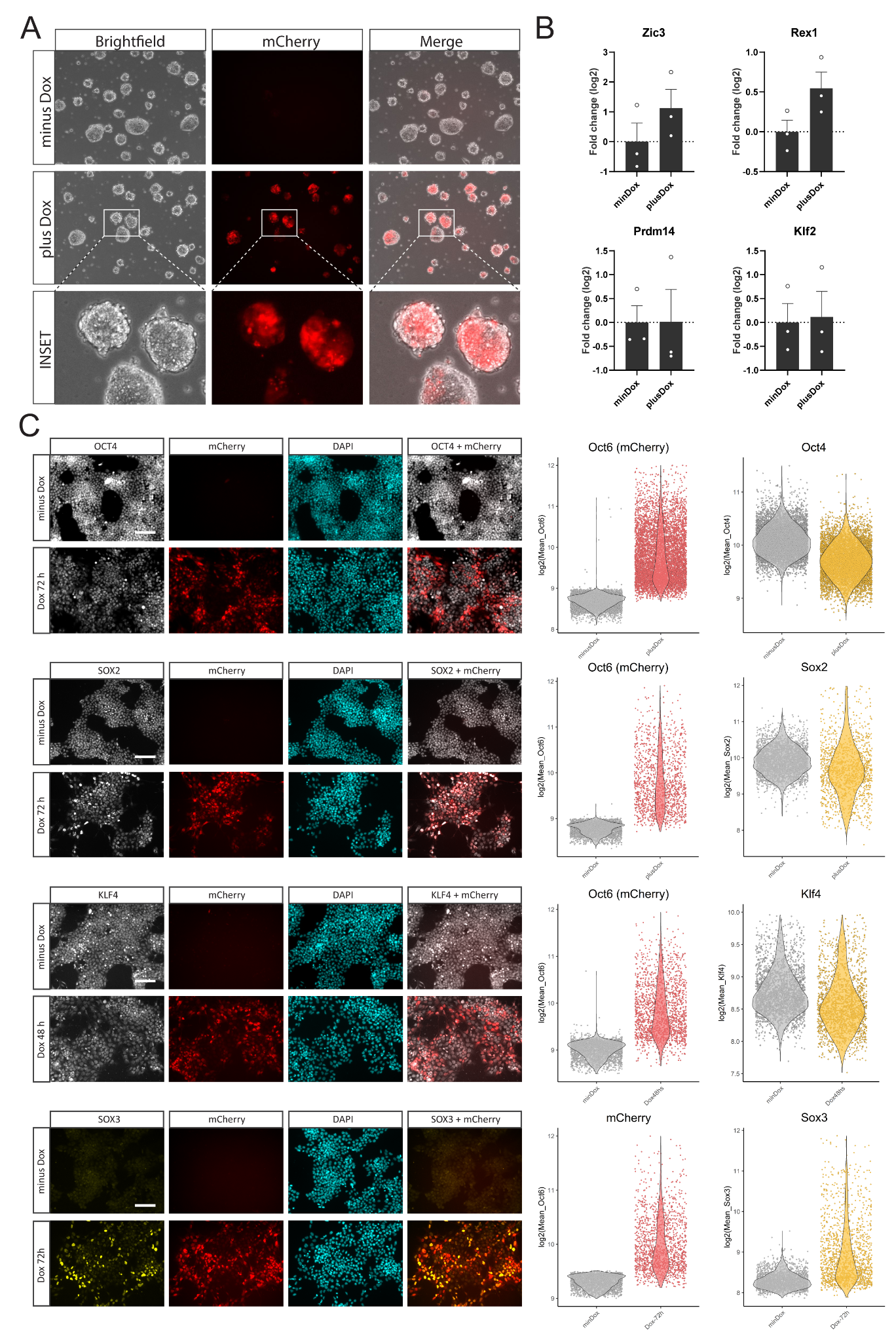


### Figure S6
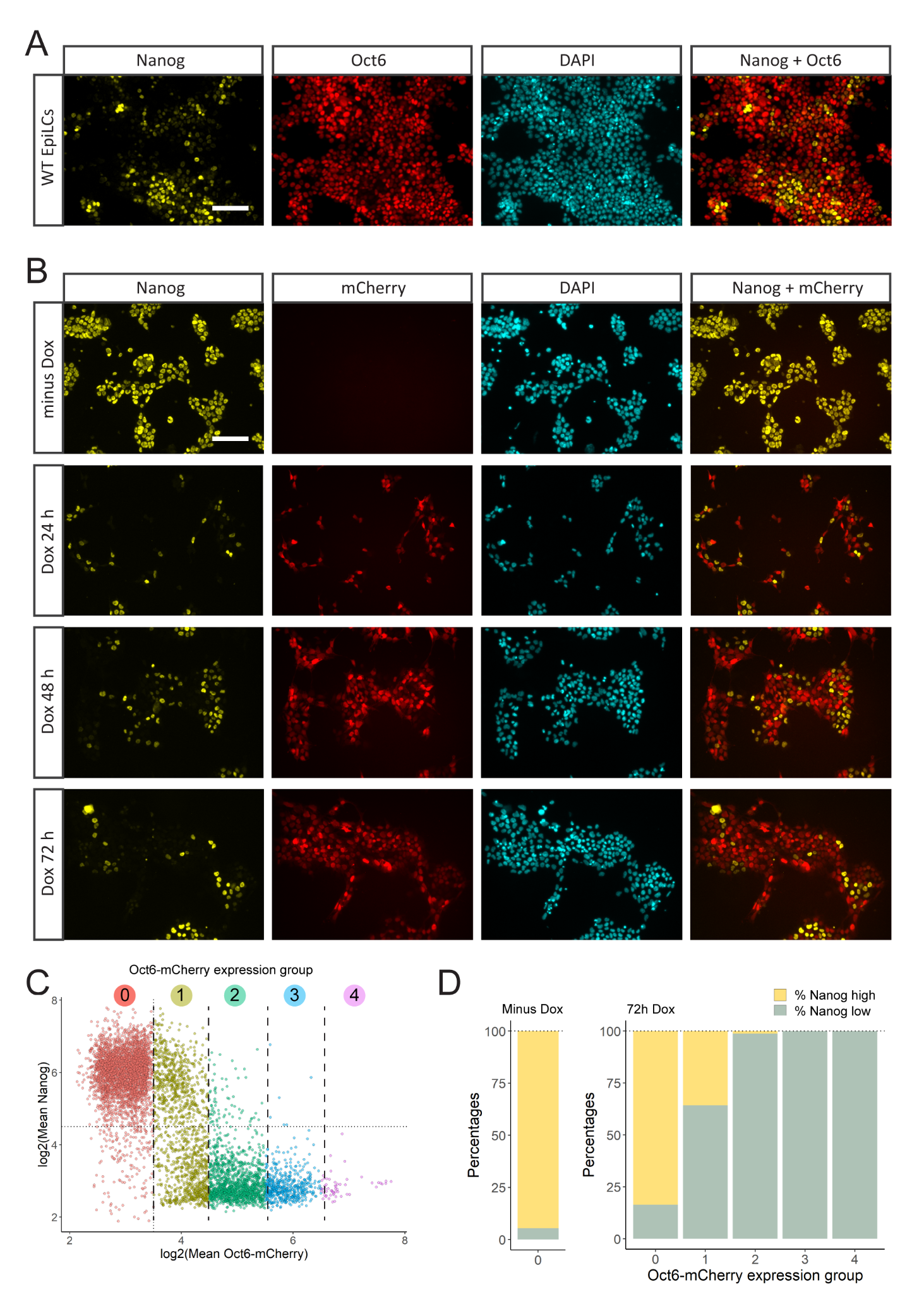


### Figure S7


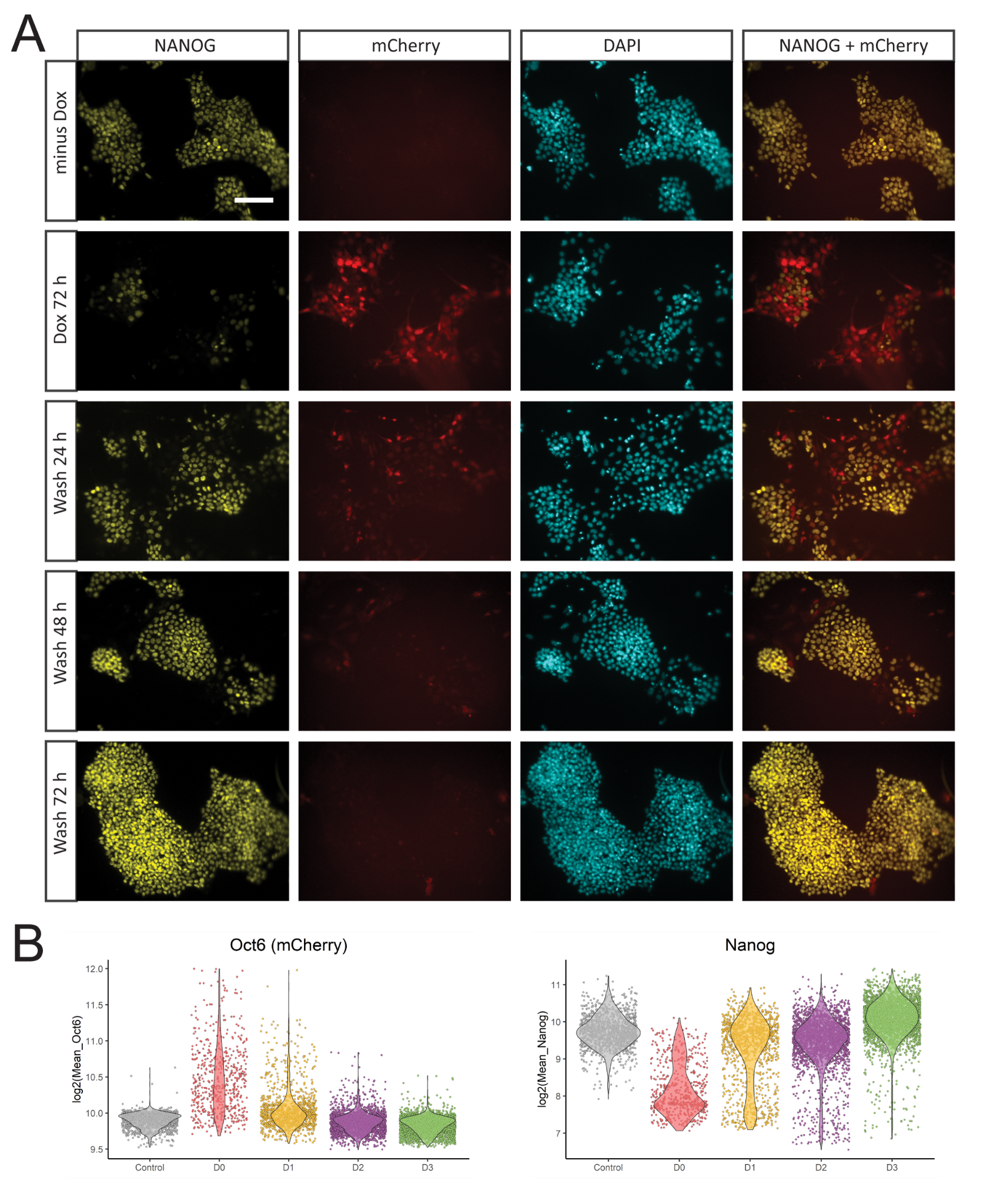


### Figure S8

###
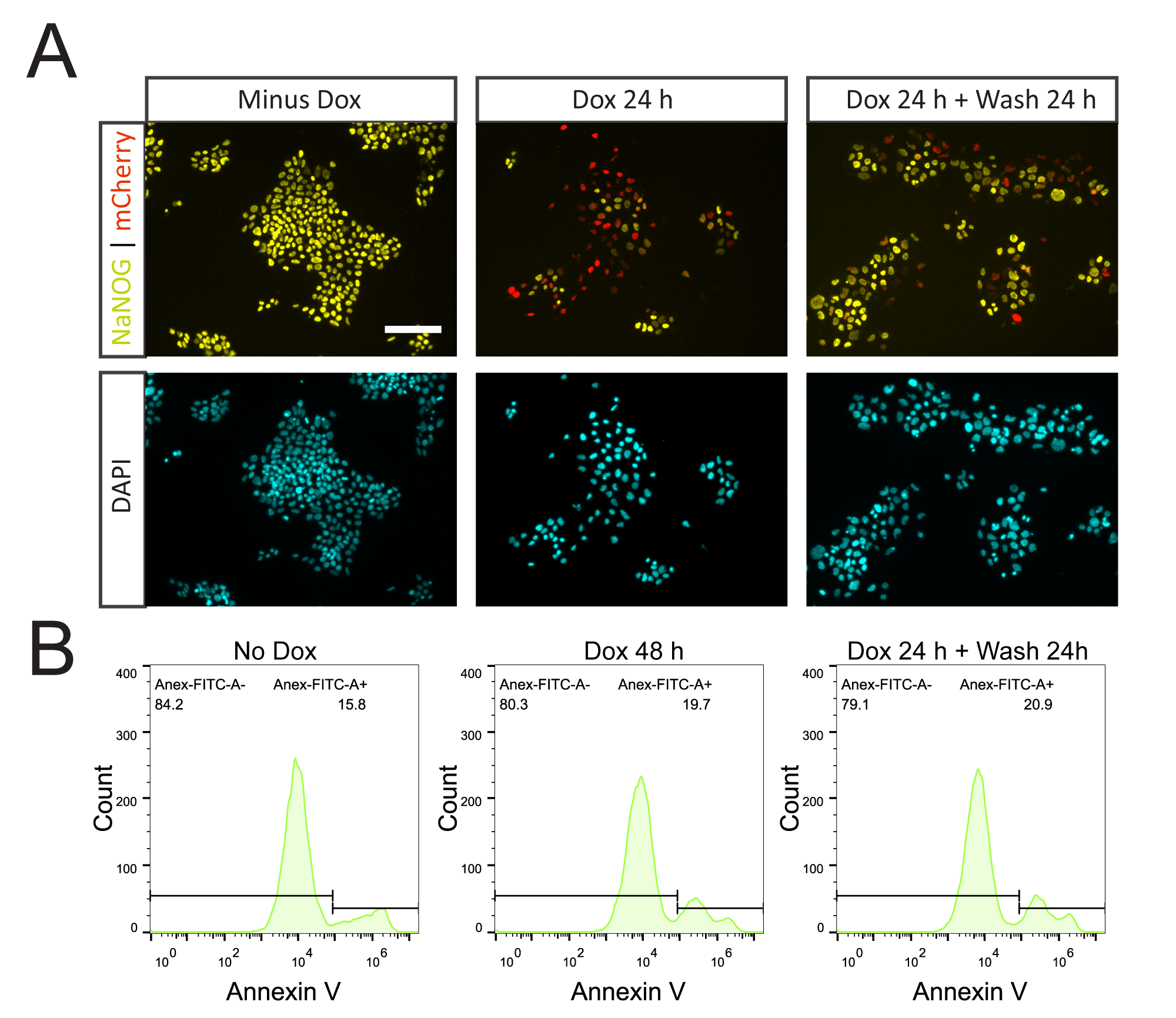


### Figure S9

**
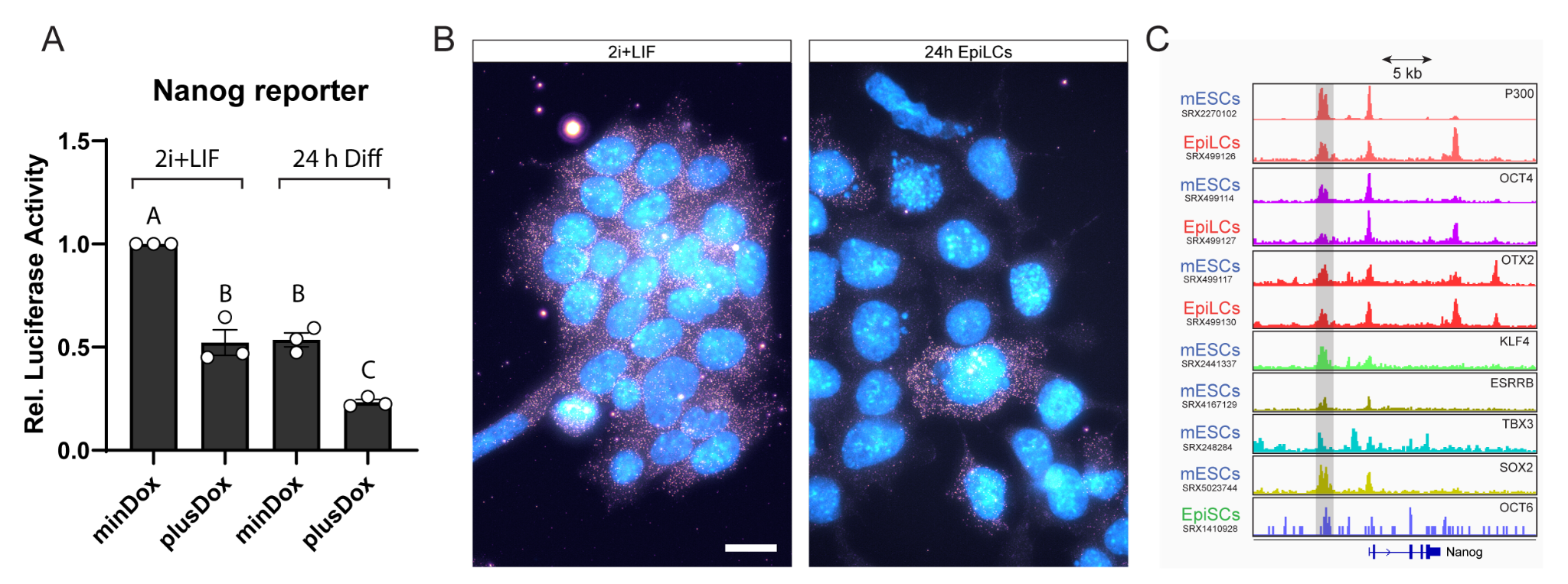
**

#

# Supplementary Figure Legends

**Figure S1. Expression of TFs induced during EpiLC differentiation**

(A) Expression of TFs induced during the differentiation of EpiLCs. Re-analysis of RNA-seq data of Yang et al [(Yang et al., 2019)](https://www.zotero.org/google-docs/?OPCUTx). (B) Representative immunofluorescence of OCT6 in undifferentiated cells and 24 or 48 h EpiLCs. Scale bar: 100 µm.

**Figure S2. Binding of pluripotency TFs and histone marks at the Oct6 promoter**

Evaluation of the binding of OCT4, OTX2, H3K27ac, H3K4me1, NANOG, and ESRRB at the *Oct6* locus based on previously published ChIP-seq experiments [(Atlasi et al., 2019; Bleckwehl et al., 2021; Buecker et al., 2014; Chen et al., 2018; Narita et al., 2021; Yang et al., 2019)](https://www.zotero.org/google-docs/?dFr262). Two CRE were identified. CRE#2 is bound by the four TFs in mESCs. OTX2 and OCT4 binding is extended to CRE#1 in EpiLCs, which also shows the topology of active enhancer marks H3K27ac and H3K4me1. The respective SRA experiment IDs are indicated on the left.

**Figure S3. Generation and validation of Oct6 KO mESCs.**

(A) DNA sequencing of the *Oct6* locus for WT 46C cells and for “Clon I” shows that the latter contains a homozygous insertion of an adenine at the Cas9 cutting site. This insertion generates a change in the open reading frame of Oct6 modifying the amino acid sequence starting from amino acid 22 and generating a premature stop codon. (B) Brightfield images showing the similar morphology of WT and *Oct6* KO mESCs cultured in 2i+LIF. (C) Immunofluorescence of the pluripotency markers OCT4, SOX2, NANOG, and KLF4 showing similar expression in WT vs Oct6 KO cells maintained in 2i+LIF. Scale bar: 100 µm. (D) Neural precursor differentiation was evaluated by flow cytometry analysis of the Sox1-GFP reporter. Comparison of WT and Oct6 KO 46C cells on day 0 (2i+LIF) or at day 6 of neural differentiation. *Left*, flow cytometry plots of a representative biological replicate. *Right*, percentage of Sox1-GFP+ neural precursors at day 6 for WT and Oct6 KO cells for three biological replicates. (E) Examples of DE gene expression from the RNA-seq results. (F) RT-qPCR analysis of E-Cadherin and N-Cadherin in WT and Oct6 KO EpiLCs. Results are as presented as mean ± SEM for three independent replicates.

**Figure S4. Binding of OCT6 to the genome in EpiSCs.**

(A) Analysis of DE genes of the RNA-seq data comparing WT vs Oct6 KO EpiLCs that contain OCT6 binding peaks in EpiSCs based on Matsuda et al. 114 out of 292 DE genes contained OCT6 binding peaks, suggesting a possible direct regulation by this TF. To obtain the random distribution of OCT6 binding among 292 randomly selected genes we followed a bootstrap strategy with 10.000 iterations. (B) OCT6 binding peaks in EpiSCs in example DE genes from our RNA-seq experiment.

**Figure S5. Overexpression of OCT6 in undifferentiated Oct6 KO cells**

(A) Overexpression of the mCherry fluorescent protein (alone) after Dox treatment did not induce any morphological changes as cells presented the typical dome shape with no cell protrusions. (B) RT-qPCR analysis of *Zic3, Rex1, Prdm14*, and *Klf2* in Oct6-P2A-mCherry cells maintained in 2i+LIF with or without Dox treatment. Results are as presented as mean ± SEM for three independent replicates. (C) Quantitative immunofluorescence of OCT4, SOX2, KLF4, and SOX3 in Oct6-P2A-mCherry cells maintained in 2i+LIF untreated or Dox treated for 24, 48, and 72 h. Panels on the left show representative immunofluorescence images. The right charts show the quantifications for mCherry and the selected TFs. Scale bar: 100 µm.

**Figure S6. OCT6 and NANOG are expressed in a mutually exclusive manner**

(A) Immunofluorescence analysis of OCT6 and NANOG in WT 48h EpiLCs showing mutually exclusive expression in normal conditions. Scale bar: 100 µm. (B) Inducible overexpression of Oct6-P2A-mCherry in Oct6 KO cells maintained in 2i+LIF shows that Oct6 represses the expression of Nanog. Cells were either untreated or treated with Dox for 24, 48, and 72 h. Scale bar: 100 µm. (C) Correlation between Oct6-P2A-mCherry and NANOG nuclear intensity expression in untreated or Dox-treated cells in 2i+LIF after immunostaining experiments. The circles show the mean fluorescence for individual cells. Cells were separated into different expression groups according to mCherry fluorescence, with each group displaying roughly 2-fold expression compared to the previous one. (D) Percentage of Nanog positive and Negative cells in each of the indicated groups in (C).

**Figure S7. Repression of NANOG by OCT6 overexpression is reversible**

(A) Oct6-P2A-mCherry cells maintained in 2i+LIF were untreated (minDox) or Dox treated for 72 hours and analyzed for mCherry and Nanog expression. Additionally, 72 h Dox treated cells were released from Dox induction and cultured for an additional 24, 48, or 72 h after washing and further evaluated for mCherry and Nanog expression. Representative images are shown. Scale bar: 100 µm. (B) Quantification of nuclear mCherry and NANOG signal of the experiment in A. mCherry fluorescence was rapidly reduced after Dox release. Nanog expression after Dox treatment is restored to similar levels as in untreated cells, showing that its repression by OCT6 is reversible.

**Figure S8. Reversion of OCT6 expression does not induce an increase in apoptosis levels**

(A) OCT6-P2A-mCherry cells maintained in 2i+LIF were untreated (minDox), treated with Dox for 48 hours, or treated with Dox for 24 hours and released from Dox for another 24 hours. Cells were analyzed for mCherry and NANOG expression by immunofluorescence to validate NANOG re-expression after Dox wash. Scale bar: 100 µm. (B) Analysis of apoptosis levels by Annexin V staining and flow cytometric analysis of the treatments indicated in (A).

**Figure S9. Single-molecule Nanog RNA fish and 3’UTR analysis of translational repression**

(A) Analysis of the Nanog5P luciferase reporter. mESCs were co-transfected with Nanog5P-Luc and ePB-HA-Oct6-P2A-mCherry plasmids, maintained in either undifferentiated conditions or set to differentiate for 24 h, in the presence or absence of Dox. Firefly luminescence was assessed to evaluate Nanog promoter activity. The plot shows normalized luminescence levels relative to undifferentiated mESCs without Dox. Results are presented as mean ± SEM for three independent replicates. Significance between groups was analyzed by linear mixed models (LMM) and indicated with different letters. (B) Single-molecule RNA fish of Nanog of cells in 2i+LIF or after 24 h of EpiLCs induction. Individual Nanog mRNA transcripts can be observed in the cell’s cytoplasm. *Nanog* transcripts are absent in the majority of the cells after 24 h of differentiation. Nuclei were stained with DAPI (cyan). Scale bar: 20 µm. (C) Binding of different TFs at the Nanog locus based on previously published ChIP-seq experiments. The respective SRA experiment IDs are indicated on the left.

#

#

#

#

# Supplementary Tables

Table S1: DEGs RNA-seq

Table S2: GO terms

Table S3: Oligonucleotides

Table S4: antibodies

Table S5: Nanog smRNA FISH probes

#

# Bibliography

[Atlasi, Y., Megchelenbrink, W., Peng, T., Habibi, E., Joshi, O., Wang, S.-Y., Wang, C., Logie, C., Poser, I., Marks, H., Stunnenberg, H.G., 2019. Epigenetic modulation of a hardwired 3D chromatin landscape in two naive states of pluripotency. Nat. Cell Biol. 21, 568–578. https://doi.org/10.1038/s41556-019-0310-9](https://www.zotero.org/google-docs/?IUoMt6)

[Bleckwehl, T., Crispatzu, G., Schaaf, K., Respuela, P., Bartusel, M., Benson, L., Clark, S.J., Dorighi, K.M., Barral, A., Laugsch, M., van IJcken, W.F.J., Manzanares, M., Wysocka, J., Reik, W., Rada-Iglesias, Á., 2021. Enhancer-associated H3K4 methylation safeguards in vitro germline competence. Nat. Commun. 12, 5771. https://doi.org/10.1038/s41467-021-26065-6](https://www.zotero.org/google-docs/?IUoMt6)

[Buecker, C., Srinivasan, R., Wu, Z., Calo, E., Acampora, D., Faial, T., Simeone, A., Tan, M., Swigut, T., Wysocka, J., 2014. Reorganization of enhancer patterns in transition from naive to primed pluripotency. Cell Stem Cell 14, 838–53. https://doi.org/10.1016/j.stem.2014.04.003](https://www.zotero.org/google-docs/?IUoMt6)

[Chen, A.F., Liu, A.J., Krishnakumar, R., Freimer, J.W., DeVeale, B., Blelloch, R., 2018. GRHL2-Dependent Enhancer Switching Maintains a Pluripotent Stem Cell Transcriptional Subnetwork after Exit from Naive Pluripotency. Cell Stem Cell 1–13. https://doi.org/10.1016/j.stem.2018.06.005](https://www.zotero.org/google-docs/?IUoMt6)

[Narita, T., Ito, S., Higashijima, Y., Chu, W.K., Neumann, K., Walter, J., Satpathy, S., Liebner, T., Hamilton, W.B., Maskey, E., Prus, G., Shibata, M., Iesmantavicius, V., Brickman, J.M., Anastassiadis, K., Koseki, H., Choudhary, C., 2021. Enhancers are activated by p300/CBP activity-dependent PIC assembly, RNAPII recruitment, and pause release. Mol. Cell 81, 2166-2182.e6. https://doi.org/10.1016/j.molcel.2021.03.008](https://www.zotero.org/google-docs/?IUoMt6)

[Yang, P., Humphrey, S.J., Cinghu, S., James, D.E., Mann, M., Yang, P., Humphrey, S.J., Cinghu, S., Pathania, R., Oldfield, A.J., 2019. Multi-omic Profiling Reveals Dynamics of the Phased Progression of Pluripotency Article Multi-omic Profiling Reveals Dynamics of the Phased Progression of Pluripotency. Cell Syst. 8, 427-445.e10. https://doi.org/10.1016/j.cels.2019.03.012](https://www.zotero.org/google-docs/?IUoMt6)
